# Downward income mobility among individuals with poor initial health is linked with higher cardiometabolic risk

**DOI:** 10.1093/pnasnexus/pgac012

**Published:** 2022-03-09

**Authors:** Grzegorz Bulczak, Alexi Gugushvili

**Affiliations:** Institute of Philosophy and Sociology, Polish Academy of Sciences, Nowy Świat 72, 00-330, Warsaw, Poland; Faculty of Management,Gdynia Maritime University, 81-87 Morska, 81-225 Gdynia, Poland; Institute of Philosophy and Sociology, Polish Academy of Sciences, Nowy Świat 72, 00-330, Warsaw, Poland; Department of Sociology and Human Geography, Harriet Holters hus, Moltke Moesvei 31, 0851, University of Oslo, Oslo, Norway; Nuffield College,University of Oxford, Oxford, UK

**Keywords:** income mobility, cardiometabolic risk, add health, the United States

## Abstract

The effects of socioeconomic position (SEP) across life course accumulate and produce visible health inequalities between different socioeconomic groups. Yet, it is not well-understood how the experience of intergenerational income mobility between origin and destination SEP, *per se*, affects health outcomes. We use data from the National Longitudinal Study of Adolescent to Adult Health collected in the United States with the outcome measure of cardiometabolic risk (CMR) constructed from data on LDL Cholesterol, Glucose MG/DL, C-reactive protein, systolic and diastolic blood pressure, and resting heart rate. Intergenerational income mobility is estimated as the difference between Waves 1 and 5 income quintiles. Diagonal reference models are used to test if intergenerational income mobility, net of origin and destination income quintile effects, is associated with CMR. We find that individuals in the lowest and the highest income quintiles have, respectively, the highest and the lowest CMR; both origin and destination income quintiles are equally important; there are no significant overall income mobility effects for different gender and race/ethnicity groups, but downward income mobility has negative health implications for individuals with poor initial health. We conclude that downward income mobility can increase inequalities in CMR in the United States by worsening the health of those who had poor health before their mobility experiences.

Significance Statement This is one of the few studies conducted on the implications of intergenerational income mobility on cardiometabolic risk (CMR) which refers to the cumulative burden of chronic stress and adverse life events on individuals’ health. Using diagonal reference models and the restricted version of the United States National Longitudinal Study of Adolescent to Adult Health (with the outcome measure constructed from data on LDL Cholesterol, Glucose MG/DL, C-reactive protein, systolic and diastolic blood pressure, and resting heart rate), we find that downward income mobility is associated with higher CMR but only among adults who had poor health before their mobility experiences.

## Introduction

A growing body of evidence indicates that socioeconomic position (SEP) in adult life is causally linked with a wide range of health outcomes (1–4). In addition, the impact of early-life conditions, i.e. parental SEP, on later-life health, often referred to as the “long arm” of childhood (dis)advantage, is also well-established (5–7). It is known that the effects of one's SEP across consecutive stages of life accumulate and produce visible health inequalities between different socioeconomic groups (8–10).

Nonetheless, it is not well-understood how the experience of intergenerational social mobility between origin and destination SEP, *per se*, affects health outcomes. Depending on the direction of social mobility, it may improve or worsen individuals’ health. On the one hand, an upward social mobility experience could result in an increased sense of control, gratitude, and satisfaction with life leading to better physiological well-being (11–14). Further, recent studies provide evidence that upward social mobility has the potential to overcome childhood disadvantages for a number of physical health measures such as functional somatic symptoms, walking speed, and lung function (7, 15). In turn, downward social mobility can be a disruptive life experience leading to psychological distress and worse mental health outcomes (16–19). Others find that downward social mobility is associated with worse physical health shown in scores on a multisystem index of physiological functioning (8).

The choice of SEP measures is important for understanding both trends in social mobility and its consequences for health (20–22). A common approach used in past research, especially in the United States, is to focus on social mobility measures based on educational or occupational attainment or a combination of these SEP indicators (22–24). Some recent studies suggest, however, that income is more strongly linked with individuals’ health than other measures of SEP (25, 26). Yet, there are only a handful of studies examining the role of intergenerational income mobility in determining health outcomes. An ecological study for the United States finds a positive link between county-level income mobility and life expectancy (27). Another study reveals that upward income mobility is linked with better mental health, but it also leads to higher metabolic syndrome rates (28). A quasi-experimental research from Japan shows that, counterintuitively, upward income mobility may have negative health implications for individuals (29). Others find no link between income mobility and health (30).

There might be at least 3 important reasons why existing studies find inconclusive results. First, scholars use various statistical approaches, some of which produce contradictory or unreliable estimates of mobility effects, net of the effects of origin ,and destination SEP (31). Second, the timing of income mobility and the age at which the destination SEP and health outcomes are measured may be important, with the mobility effects taking place primarily in the first half of life (32). Third, the described and related studies usually do not account for individuals’ health before their social mobility experiences. This is problematic because health in childhood *via* the process of selection might be linked to adulthood health, individuals’ social mobility prospects, and their actually attained SEP (33).

Exploring the health implications of income mobility in the United States might be also relevant in relation to individuals’ gender and race/ethnicity. A growing body of evidence suggests that structural sexism and structural racism are among the central social determinants of health and health inequalities in the United States (34–37). A myriad of mutually reinforcing systems of education, employment, living conditions, welfare services, credit and mortgage, media, health care, and criminal justice not only affect individuals’ likelihood to experience income mobility, but they can also mean the health-related costs and benefits of experiencing intergenerational mobility are unequally distributed across gender and race/ethnicity groups (38–40). Selected empirical evidence, for instance, suggests that the experience of upward educational mobility among members of minorities from disadvantaged backgrounds in the United States improves their psychological well-being but compromises their physical health (41). Yet, to our knowledge, it has not been tested whether intergenerational income mobility has varying implications for individuals’ across gender and race/ethnicity groups.

To address the described methodological and substantive research gaps, in this study we analyze intergenerational income mobility in the United States among a representative sample of adults aged 42 or below, using Sobel's diagonal reference models (DRMs), developed to examine the role of social mobility, net of SEP effects (42). The main conclusion of our study is that downward income mobility among adults who had poor health before their mobility experiences is associated with higher cardiometabolic risk (CMR), a reliable health measure that we describe in detail below.

## Methods

### Data

We used data from Waves 1 and 5 of the National Longitudinal Study of Adolescent to Adult Health, Add Health, collected in the United States, respectively, in 1994–1995 and 2016–2018 (43). The dataset has been previously used for exploring the health consequences of social mobility and contains rich information for constructing indicators of income mobility as well as data on objective health outcome measures (22, 24). Our analysis was restricted to individuals that had complete biomarker data based on Wave 5 blood test (*n* = 5,276). The average age of respondents at the time of the survey was 37 (min–max: 32–42). Due to missing data, primarily for Wave 1 income variable (17% of the sample), we used multiple imputation to preserve the sample size by employing the Multiple Imputation by Chained Equations (MICE) procedure (44). All the analyses reported in the main text are based on the imputed data, while estimations using a listwise deletion procedure provided consistent results and are reported in Tables S1 and S2 (Supplementary Material). For this analysis we used a restricted sample from the Add Health dataset which cannot be shared.

### CMR

We aimed to construct a health outcome measure that is most likely to reliably capture the impact of origin and destination SEP and income mobility. Because our sample consists of relatively young adults with overall good levels of health, it was important to analyze a health measure sensitive enough to provide sufficient variation among participants. One such health measure is CMR, which has the potential not only to reveal current health problems shaped by lifetime exposures of the body to adverse events, but also to act as an early warning signal for future health problems (45–47). An illustrative example is offered by a recent study of middle-aged individuals from the United Kingdom examining the association between 1 of the components of CMR, LDL cholesterol, and the risk of developing dementia more than 10 years later (48).

Following past research, we constructed an aggregated health measure, CMR, based on Wave 5 blood test and home examination data (49–51). More specifically, the following 6 components were used to construct the main health outcome variable: (1) Lipid—LDL Cholesterol; (2) Glucose—Glucose MG/DL; (3) Inflammation—C-reactive protein (CRP); and (4) Cardiovascular—(4.1) systolic blood pressure, (4.2) diastolic blood pressure, and (4.3) resting heart rate. These biomarkers are significantly associated with the likelihood of experiencing vascular events and developing diabetes. We z-transformed each of these components and calculated the mean score. Lastly, we also z-transformed the score to obtain the final CMR measure. Descriptive statistics of the measures used to construct the CMR variable are presented in Table S3 (Supplementary Material).

### Origin, destination, and intergenerational income mobility

We used Add Health's Wave 1 parental household income data, divided into quintiles, as the individuals’ origin SEP measure. The destination SEP measure is derived from Wave 5 personal income, also divided into quintiles. To account for individuals’ household size at Waves 1 and 5, we adjusted household and personal income based on equivalence scales (income/(household size)^0.5^) used in the previous research for the United States (52, 53). We estimated individuals’ intergenerational income mobility as the difference between Waves 1 and 5 income quintiles. If we observed no difference in quintile position between the waves, individuals were classified as immobile, and as downwardly or upwardly mobile in cases where parental income quintile position was, respectively, higher or lower in comparison to individuals’ income quintile. The constructed intergenerational income mobility measure takes values from −4 to 4. As moving up or down by 2 or more income quintiles was relatively rare in our sample, we created 2 sets of income mobility trajectories for short- (one step) and long-range (2 or more steps) mobility to ensure a comparable number of individuals in different mobility trajectories and to ease the interpretation of the results. Figure S1 (Supplementary Material), depicts the symmetrical distribution of the constructed income mobility variable.

### Wave 1 health, gender, and race/ethnicity

We accounted for adolescents’ health prior to mobility experiences by introducing a binary “poor health” indicator, which is equal to 1 if self-reported health was rated by participants as below very good in Wave 1. We rely on self-rated health because there is no corresponding data to construct a matching CMR measure in Wave 1. Self-rated health among adolescents was shown to be a valid indicator of health in the United States (54). We also constructed 2 other early health indicators; first, a binary variable for chronic health problems, which is equal to 1 if respondents answered “yes” to any of the 4 following questions: “Do you have difficulty using your hands, arms, legs, or feet because of a permanent physical condition?,” “Do you have a permanent physical condition involving a heart problem?,” “Do you have a permanent physical condition involving asthma?,” and “Do you have a permanent physical condition involving other breathing difficulties?”; and second, a binary variable for being obese (having body mass index equal to 30 or above).

Having chronic health problems and being obese are important aspects of individuals’ initial health, but their prevalence in Wave 1 was much lower than self-rated poor health, which also captures the broader aspects of well-being. Therefore, we use self-rated poor health as the main indicator of initial health but we still account for individuals’ chronic health problems and obesity in Wave 1 in our statistical models. All estimated models account for individuals’ age and gender. In the final models, we include individuals’ race/ethnicity variables, consisting of Hispanic, (non-Hispanic) Black, (non-Hispanic) other, or Asian categories, (non-Hispanic) Whites being the reference group. Table S4 (Supplementary Material) presents descriptive statistics for the selected variables.

### Statistical approach

We used DRMs to test if intergenerational income mobility, net of origin, and destination income quintile effects, is associated with CMR (31, 42). The DRM approach is commonly used in social mobility research to overcome the problem of multicollinearity arising due to mobility being directly estimated from origin and destination SEP (49, 55, 56, 5774). DRMs are employed to examine not only the role of social mobility in explaining the variation in various outcome measures but also the relative importance of origin and destination SEP. In tables with DRM estimates, we present the main results starting with immobile individuals for whom coefficients show CMR differences between 5 income quintiles. This is followed by the presentation of origin weight which takes a value between 0 and 1. The destination weight in a DRM is equal to 1 − the origin weight. For example, an origin weight equal to 0.3 indicates that an individual's own position (destination) is relatively more important in determining the outcome measure (1 − 0.3 = 0.7), the level of CMR in our case. We capture income mobility effects by including mobility parameters, interpretable as in conventional regression models, for long- and short-range downward and upward income mobility. To test if the effect of social origin on CMR and the health implications of income mobility varied by the individuals’ gender, race/ethnicity, and initial health, we fit models with interaction terms between the described parameters.

## Results

### Income mobility trajectories and CMR

**Fig. 1.**
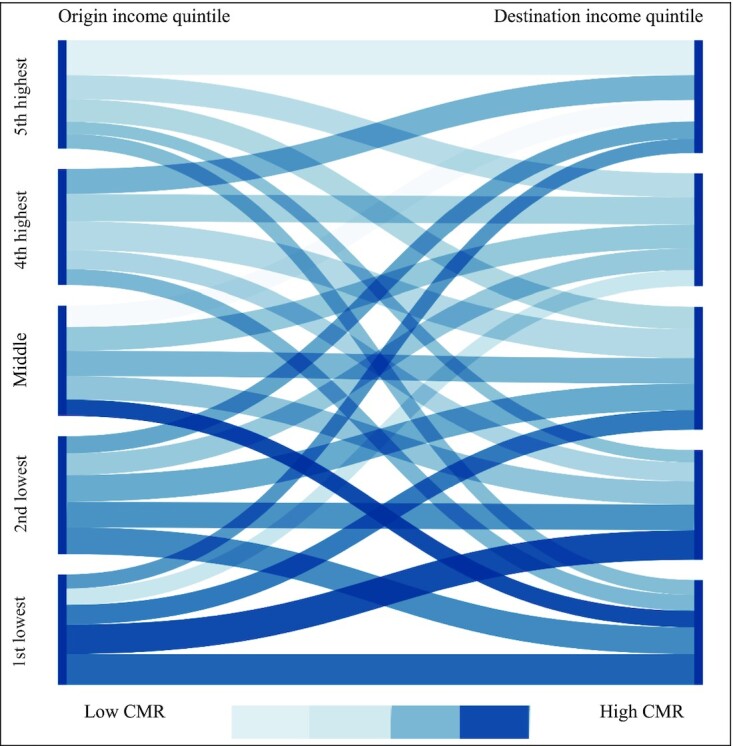
Income mobility and CMR levels. Notes: higher flows’ color saturations indicate higher mean CMR levels. The width of the flows is proportional to the flow quantity, *n* = 5,276.

Figure 1 shows a Sankey diagram presenting different income mobility trajectories and corresponding levels of CMR. On the one hand, the width of each flow reflects the relative number of individuals in each group. It can be seen that very few individuals moved from the first or lowest income quintile, bottom left, to the fifth or highest income quintile, top right. On the other hand, the flow saturation shows CMR levels, and this suggests that high income is associated with better health. This is most clearly visible in the case of immobile individuals who stayed in the same income quintile as their parents. Among all mobility trajectories, the lowest CMR level (the lightest saturation) is observed among individuals that moved up the income ladder from the middle to the highest income quintile, while those moving down by 2 steps to the lowest income group record the highest CMR (the highest saturation). The Sankey diagram highlights the complexities associated with examining the effects of origin, destination ,and income mobility on individuals’ health. To disentangle these effects, to estimate the relative weight of origin and destination income quintile for CMR, and also to account for the samples’ demographic and initial health composition, we turn to the DRM approach.

### Estimating position and mobility effects

Confirming the descriptive findings, Model 1 in Table 1 shows that immobile individuals in the lowest income quintile have much higher CMR (0.34, 95% confidence interval (CI95): 0.26, 0.43) than individuals in other immobile income categories. Similarly, the higher the income the better the health, with those in the highest quintile having the lowest CMR (−0.24, CI95: −0.32, −0.15). Origin weight (0.52, CI95: 0.38, 0.67) indicates that the destination plays an equally important role in determining CMR (the destination weight is: 1.00 − 0.52 = 0.48). Considering the large CIs of these weight parameters in Models 1–4, we can state that both origin and destination income quintiles are equally important for individuals’ health. As expected, age is significantly and positively associated with CMR. We also observe that males have higher CMR.

**Table 1.** Income mobility and CMR levels, point estimates from DRMs.

	Model 1		Model 2		Model 3	
	*β*	[CI95]	*β*	[CI95]	*β*	[CI95]
Intercept	0.99***	[0.97, 1.01]	0.99***	[0.97, 1.01]	0.98***	[0.96, 1.00]
*Immobile income quintiles*						
1st lowest	0.34***	[0.26, 0.43]	0.34***	[0.25, 0.43]	0.31***	[0.22, 0.40]
2nd lowest	0.12**	[0.03, 0.21]	0.12**	[0.03, 0.21]	0.10*	[0.01, 0.20]
Middle	–0.06	[−0.14, 0.03]	–0.06	[−0.14, 0.03]	–0.05	[−0.13, 0.03]
4th highest	–0.17***	[−0.26, −0.08]	–0.17***	[−0.26, −0.08]	–0.15**	[−0.23, −0.06]
5th highest	–0.24***	[−0.32, −0.15]	–0.24***	[−0.32, −0.15]	–0.22***	[−0.30, −0.13]
Origin weight	0.52***	[0.38, 0.67]	0.62***	[0.34, 0.91]	0.60***	[0.27, 0.93]
Age	0.02*	[0.00, 0.04]	0.02*	[0.01, 0.04]	0.02**	[0.01, 0.04]
Sex (male = 1)	0.35***	[0.28, 0.41]	0.35***	[0.28, 0.41]	0.36***	[0.30, 0.42]
*Income mobility*						
Long-range downward	–––––	–––––––––	0.04	[−0.05, 0.13]	0.05	[−0.04, 0.14]
Short-range downward	–––––	–––––––––	0.01	[−0.08, 0.10]	0.01	[−0.07, 0.10]
Long-range upward	–––––	–––––––––	–0.07	[−0.23, 0.08]	–0.06	[−0.22, 0.10]
Short-range upward	–––––	–––––––––	0.03	[−0.10, 0.16]	0.03	[−0.09, 0.16]
*Race/ethnicity (ref. = White non-Hispanic)*						
Hispanic	–––––	–––––––––	–––––	–––––––––	–0.10*	[−0.19, −0.01]
Black non-Hispanic	–––––	–––––––––	–––––	–––––––––	0.30***	[0.22, 0.38]
Asian	–––––	–––––––––	–––––	–––––––––	–0.13	[−0.26, 0.01]
Other	–––––	–––––––––	–––––	–––––––––	–0.27	[−0.62, 0.09]
Adjusted *R*^2^	0.047		0.047		0.065	
Observations	5,276		5,276		5,276	

*Note:* *P < 0.05, **P < 0.01, and ***P < 0.001.

In Model 2, upward and downward income mobility parameters, both short- and long-range, are introduced. Although none of these are statistically significant, the importance of origin weight increases. In Model 3 we account for individuals’ race/ethnicity, an important determinant of CMR (58). Our results suggest that Black individuals have markedly higher and Hispanics slightly lower CMR in comparison to White non-Hispanic individuals. The inclusion of the race/ethnicity variable has little effect on the health gradient among immobile individuals or on the value of origin weight (0.60, CI95: 0.27, 0.93).

### Gender and race/ethnicity interactions

Next, we explore interaction effects between the origin weight, mobility parameters, and individuals’ main demographic characteristics to test if the identified associations are affected by individuals’ gender and race/ethnicity. Table 2 shows the estimates only for the interaction terms. None of these interactions are statistically significant, which suggests that the importance of social origin and social mobility effects do not vary by individuals’ main demographic characteristics.

**Table 2.** Origin weight and mobility interactions with gender and race/ethnicity, point estimates from DRMs.

		Income mobility			
	Origin weight	Short-range upward	Long-range upward	Short-range downward	Long-range downward
Male	−0.03	−0.02	0.03	0.01	0.07
	[−0.31, 0.25]	[−0.21, 0.17]	[−0.14, 0.20]	[−0.10, 0.12]	[−0.09, 0.23]
Hispanic	−0.10	−0.02	−0.03	−0.03	0.03
	[−0.50, 0.31]	[−0.30, 0.25]	[−0.28, 0.22]	[−0.33, 0.27]	[−0.25, 0.30]
Black non-Hispanic	−0.11	−0.06	0.05	0.02	0.15
	[−0.44, 0.22]	[−0.28, 0.17]	[−0.16, 0.25]	[−0.22, 0.25]	[−0.05, 0.36]
Asian	−0.44	−0.07	−0.17	−0.07	0.04
	[−1.09, 0.22]	[−0.49, 0.35]	[−0.55, 0.21]	[−0.59, 0.44]	[−0.38, 0.45]
Other race/ethnicity	−0.61	−0.82	−0.68	−0.21	−0.54
	[−2.23, 1.01]	[−1.97, 0.32]	[−1.90, 0.53]	[−1.25, 0.84]	[−1.60, 0.51]

*Notes:* *P < 0.05, **P < 0.01, and ***P < 0.001. Number of observations = 5,276.

### The role of early-life health

Based on earlier research (22, 33), it is likely that individuals’ health prior to their social mobility experience affects both mobility trajectories and Wave 5 CMR. The following analysis confirms our expectations. First, we use the binary poor health variable for initial health and also test the robustness of our findings by including other early health indicators, dummy variables for chronic health problems and obesity. Model 1 in Table 3 shows that adolescents’ health is an important predictor of Wave 5 CMR. However, the impact of this variable is negligible in terms of changes in the model parameters of interest. By including interaction terms in the next 3 models, we test if the importance of social origin and social mobility on CMR differs with respect to individuals’ early-life health. The results in Model 2 indicate that the origin income quintile and the destination income quintile have similar importance for individuals with poorer Wave 1 health. In turn, Models 3 and 4 indicate that downward mobility by 2 or more quintiles has negative health implications for individuals with poor initial health. This association remains robust when individuals’ Wave 1 chronic health problems and obesity are also accounted for in Model 4.

**Table 3.** Income mobility and CMR levels, point estimates from DRMs with Wave 1 poor health interactions.

	Model 1		Model 2		Model 3		Model 4	
	*β*	[CI95]	*β*	[CI95]	*β*	[CI95]	*β*	[CI95]
Intercept	0.98***	[0.95, 1.00]	0.98***	[0.95, 1.00]	0.97***	[0.95, 1.00]	0.96***	[0.94, 0.98]
*Immobile income quintiles*								
1st lowest	0.29***	[0.20, 0.38]	0.29***	[0.21, 0.38]	0.29***	[0.20, 0.38]	0.27***	[0.18, 0.36]
2nd lowest	0.09	[−0.00, 0.19]	0.08	[−0.02, 0.17]	0.10*	[0.00, 0.19]	0.09	[−0.00, 0.18]
Middle	–0.04	[−0.13, 0.04]	–0.04	[−0.12, 0.04]	–0.05	[−0.13, 0.03]	–0.05	[−0.13, 0.04]
4th highest	–0.14**	[−0.22, −0.05]	–0.14**	[−0.23, −0.06]	–0.14**	[−0.22, −0.05]	–0.12**	[−0.21, −0.04]
5th highest	–0.20***	[−0.29, −0.11]	–0.19***	[−0.28, −0.10]	–0.20***	[−0.29, −0.11]	–0.19***	[−0.27, −0.11]
Origin weight	0.62***	[0.27, 0.97]	0.70***	[0.36, 1.04]	0.63***	[0.29, 0.97]	0.66***	[0.32, 1.01]
*Income mobility*								
Long-range downward	0.05	[−0.04, 0.13]	0.04	[−0.04, 0.13]	–0.01	[−0.10, 0.09]	–0.00	[−0.10, 0.09]
Short-range downward	0.01	[−0.07, 0.10]	0.02	[−0.07, 0.10]	0.01	[−0.10, 0.11]	0.01	[−0.09, 0.11]
Long-range upward	–0.06	[−0.22, 0.10]	–0.06	[−0.21, 0.10]	–0.07	[−0.24, 0.10]	–0.08	[−0.24, 0.08]
Short-range upward	0.03	[−0.10, 0.16]	0.04	[−0.09, 0.16]	0.05	[−0.08, 0.19]	0.05	[−0.08, 0.19]
*Wave 1 health*								
Poor self-rated health	0.22***	[0.16, 0.28]	0.22***	[0.16, 0.29]	0.19***	[0.08, 0.30]	0.13*	[0.02, 0.25]
Chronic health condition	–––––	–––––––––	–––––	–––––––––	–––––	–––––––––	0.13	[−0.07, 0.32]
Obese	–––––	–––––––––	–––––	–––––––––	–––––	–––––––––	0.48***	[0.36, 0.60]
*Interactions terms*								
Origin weights * Wave 1 health	–––––	–––––––––	–0.30	[−0.61, 0.02]	–––––	–––––––––	–––––	–––––––––
Long-range downward * Wave 1 health	–––––	–––––––––	–––––	–––––––––	0.18*	[0.01, 0.34]	0.18*	[0.02, 0.34]
Short-range downward * Wave 1 health	–––––	–––––––––	–––––	–––––––––	0.02	[−0.17, 0.22]	0.01	[−0.19, 0.21]
Long-range upward * Wave 1 health	–––––	–––––––––	–––––	–––––––––	0.03	[−0.16, 0.21]	0.05	[−0.13, 0.23]
Short-range upward * Wave 1 health	–––––	–––––––––	–––––	–––––––––	–0.08	[−0.27, 0.11]	–0.08	[−0.26, 0.11]
Adjusted *R*^2^	0.074		0.074		0.075		0.080	
Observations	5,276		5,276		5,276		5,276	

*Notes:* *P < 0.05, **P < 0.01, and ***P < 0.001. All models in Table 3 account for age, gender, and race/ethnicity variables.

### Robustness checks

In supplementary materials, we further examine the role of individuals’ Wave 5 educational and occupational attainment, but these results should be interpreted with caution as both considered variables are known to be linked with health and are also strongly associated with income. After individuals’ education or occupation (both measured in quintiles) are accounted for, Table S5 (Supplementary Material), education is considerably more important in determining CMR than occupational attainment, but this does not change the observed results in the main analysis. We also address the potential issue of CMR-related medication use. The main concern is that for individuals with an identified health problem, CMR may be lower than expected due to the use of drugs aiming to alleviate the specific health condition. Therefore, we introduce additional controls showing whether or not respondents had taken medications related to a specific health condition (anti-inflammatory, antidiabetic, antihyperlipidemic, and antihypertensive) in the previous 4 weeks before the blood test. The results, presented in Table S6 (Supplementary Material), remain robust. Lastly, the main results were based on personal income reported at Wave 5, and we rerun this analysis with household income reported in the same wave. The results, presented in Table S7 (Supplementary Material), show a steeper health gradient for the immobile groups and the stronger role of the destination position in determining CMR. Yet, we identify no major differences for income mobility and its interaction with Wave 1 health, which remains statistically significant.

## Discussion

Individuals’ SEP is a key factor shaping health disparities and both adulthood and childhood social standings are known to affect short- and long-term health, mortality, and longevity (1, 6, 59–61). Past studies on social mobility effects have mostly focused on self-reported health outcomes and educational and occupational attainment or some type of combined SEP measures as indicators of social mobility (32, 62). So far, few studies have examined the health implications of intergenerational income mobility and the role of health selection in this process. Recent research using the same data as we do in the current paper but a distinct analytical approach, finds that upward income mobility is associated with higher metabolic syndrome rates (28). Other studies also confirm that findings in the field depend not only on the choice of health outcome measures but also on how SEP and mobility indicators are operationalized (41, 63, 64). Further, individuals’ initial health is likely to affect mobility trajectories and should be carefully accounted for (22, 33).

We contribute to the previous findings by using a specialized statistical approach, DRM, income-based mobility measures, and the indicators of health before social mobility experiences take place. The findings of this study show that childhood and adulthood SEP of adult Americans, measured by the income quintile they belong to, matter to a similar extent in determining an actual health outcome, CMR. In other words, individuals’ origin and destination positions appear to be equally important for their health. We show that individuals in the lowest and the highest income quintiles have, respectively, the highest and the lowest CMR and that this pattern is robust to a number of additional tests. The latter is in line with existing evidence that the United States has major differences in health outcomes measured by individuals’ income and these disparities are partially explained by the specific features of its health care system such as high out-of-pocket spending and fragmented and poorly targeted insurance coverage (65, 66).

We find no evidence of net income mobility effects. However, after accounting for the issue of health selection, we observe that among individuals with poor initial health, downward mobility by 2 or more income quintiles is associated with higher CMR. These results are robust to alternative model specifications, including using the attained SEP based on household income and accounting for CMR-related medication use. The described findings highlight the importance of considering the health selection mechanisms in research on the health consequences of intergenerational social mobility. Our results show that some forms of downward mobility are likely to exacerbate already existing health problems and increase inequalities in health. The latter may explain, to some extent, why past research in the field produced null or inconclusive findings. Arguably, the effects of social mobility kick in only among the specific groups of individuals who are particularly vulnerable healthwise before entering their adolescent years.

In addition, we demonstrate significant gender and race/ethnicity effects on CMR, with males and Blacks having worse health than females and non-Hispanic Whites, yet we identify no significant income mobility effects with respect to individuals’ gender and race/ethnicity. Various social forces, as described by structural racism and structural sexism perspectives, strongly affect individuals’ life chances and their likelihood of experiencing downward or upward income mobility (67–69). Yet, individuals who move up or slide down in the income hierarchy do not appear to be different in terms of the health implications of intergenerational income mobility across gender and racial/ethnic groups. One explanation for this could be that upwardly mobile individuals, regardless of their gender and race/ethnicity, are positively selected by personal characteristics, which are conducive to both upward income mobility and later-life health (70, 71). On the other hand, those who experience downward income mobility can be equally disadvantaged and this adverse experience does not manifest in visible differences between different gender and race/ethnicity groups (72, 73).

Our study has its limitations. The health outcome measure, CMR, is suitable to study the health consequences of income mobility experiences but it is not ideal. In the robustness checks, we account for CMR-related medication use, yet this still leaves some scope for uncertainty with respect to the effects of different medications on this aggregate health measure. Also, because our sample consists of relatively young adults, there is the possibility that, as more health problems appear and accumulate with age, the effects of income and income mobility on health may change. Further, as our Wave 1 health measures differ from Wave 5 measures, in future research, it would be of interest to examine how accounting for initial CMR (not possible with the current data) affects the findings. These limitations indicate possible new avenues for subsequent studies using the forthcoming Add Health waves. Despite the described limitations, we conclude that downward income mobility can increase inequalities in health in the United States by worsening CMR of those who had poor health before their mobility experiences.

## Supplementary Material

pgac012_Supplemental_File

## Data Availability

The access to data underlying this article is provided by Add Health, Carolina Population Center, University of North Carolina at Chapel Hill at https://addhealth.cpc.unc.edu/. The Stata code for the presented analysis is available via Open Science Framework (https://osf.io/3t82m/) (74).
