# Supplementary material for: Downward income mobility among individuals with poor initial health is linked with higher cardiometabolic risk
Source: PNAS Nexus. 2022 Mar 9;1(1):pgac012. doi: 10.1093/pnasnexus/pgac012 (PMC9802411; doi:10.1093/pnasnexus/pgac012)
Supplement: pgac012_Supplemental_File [file pgac012_supplemental_file.docx]

**Supplementary materials**

Figure S1: Intergenerational income mobility trajectories

**Table S1:** Listwise deletion, models as in Table 1 in the main text

|  | Model 1 | | Model 2 | | Model 3 | |
| --- | --- | --- | --- | --- | --- | --- |
|  | β | [CI95] | β | [CI95] | β | [CI95] |
| Intercept | 0.97^***^ | [0.95,1.00] | 0.97^***^ | [0.95,1.00] | 0.96^***^ | [0.94,0.99] |
| *Immobile income quintiles* |  |  |  |  |  |  |
| 1^st^ lowest | 0.32^***^ | [0.21,0.43] | 0.33^***^ | [0.22,0.44] | 0.30^***^ | [0.19,0.41] |
| 2^nd^ lowest | 0.11^*^ | [0.01,0.22] | 0.11^*^ | [0.00,0.22] | 0.10 | [-0.02,0.21] |
| Middle | –0.04 | [-0.15,0.06] | –0.04 | [-0.15,0.06] | –0.03 | [-0.14,0.07] |
| 4^th^ highest | –0.18^***^ | [-0.29,-0.08] | –0.18^***^ | [-0.29,-0.07] | –0.16^**^ | [-0.27,-0.06] |
| 5^th^ highest | –0.21^***^ | [-0.31,-0.11] | –0.21^***^ | [-0.32,-0.10] | –0.20^***^ | [-0.30,-0.09] |
| Origin weight | 0.45^***^ | [0.29,0.60] | 0.58^**^ | [0.23,0.93] | 0.50^*^ | [0.11,0.88] |
| Age | 0.02 | [-0.00,0.04] | 0.02 | [-0.00,0.04] | 0.02 | [-0.00,0.05] |
| Sex (male=1) | 0.31^***^ | [0.23,0.39] | 0.31^***^ | [0.23,0.39] | 0.33^***^ | [0.25,0.41] |
| *Income mobility* |  |  |  |  |  |  |
| Long-range downward | ––––– | ––––––––– | 0.07 | [-0.06,0.19] | 0.05 | [-0.07,0.18] |
| Short-range downward | ––––– | ––––––––– | –0.02 | [-0.13,0.09] | –0.02 | [-0.13,0.09] |
| Long-range upward | ––––– | ––––––––– | –0.09 | [-0.27,0.09] | –0.06 | [-0.24,0.12] |
| Short-range upward | ––––– | ––––––––– | 0.07 | [-0.07,0.21] | 0.08 | [-0.05,0.22] |
| *Race/ethnicity (ref.= White non-Hispanic)* |  |  |  |  |  |  |
| Hispanic | ––––– | ––––––––– | ––––– | ––––––––– | –0.09 | [-0.21,0.04] |
| Black non-Hispanic | ––––– | ––––––––– | ––––– | ––––––––– | 0.31^***^ | [0.21,0.41] |
| Asian | ––––– | ––––––––– | ––––– | ––––––––– | –0.06 | [-0.26,0.13] |
| Other | ––––– | ––––––––– | ––––– | ––––––––– | –0.40 | [-0.86,0.06] |
| AIC | 6927.32 |  | 6928.91 |  | 6891.35 |  |
| BIC | 6979.67 |  | 7004.52 |  | 6990.22 |  |
| Observations | 3354 |  | 3354 |  | 3354 |  |

*Notes:* * p < 0.05, ** p < 0.01, *** p < 0.001.

**Table S2:** Listwise deletion, models as in Table 3

|  | Model 1 | | Model 2 | | Model 3 | | Model 4 | |
| --- | --- | --- | --- | --- | --- | --- | --- | --- |
|  | β | [CI95] | β | [CI95] | β | [CI95] | β | [CI95] |
| Intercept | 0.96^***^ | [0.93,0.99] | 0.96^***^ | [0.93,0.98] | 0.96^***^ | [0.93,0.98] | 0.95^***^ | [0.92,0.98] |
| *Immobile income quintiles* |  |  |  |  |  |  |  |  |
| 1^st^ lowest | 0.27^***^ | [0.16,0.38] | 0.27^***^ | [0.16,0.37] | 0.27^***^ | [0.16,0.38] | 0.27^***^ | [0.16,0.38] |
| 2^nd^ lowest | 0.09 | [-0.03,0.20] | 0.07 | [-0.02,0.17] | 0.09 | [-0.02,0.21] | 0.09 | [-0.02,0.20] |
| Middle | –0.03 | [-0.13,0.08] | –0.00 | [-0.10,0.09] | –0.03 | [-0.14,0.08] | –0.04 | [-0.14,0.07] |
| 4^th^ highest | –0.15^**^ | [-0.25,-0.04] | –0.17^***^ | [-0.27,-0.08] | –0.15^**^ | [-0.25,-0.04] | –0.13^*^ | [-0.24,-0.03] |
| 5^th^ highest | –0.19^***^ | [-0.29,-0.08] | –0.17^**^ | [-0.27,-0.07] | –0.19^***^ | [-0.29,-0.08] | –0.19^***^ | [-0.29,-0.08] |
| Origin weight | 0.51^*^ | [0.09,0.93] | 0.70^***^ | [0.29,1.11] | 0.53^*^ | [0.11,0.95] | 0.61^**^ | [0.18,1.04] |
| *Income mobility* |  |  |  |  |  |  |  |  |
| Long-range downward | 0.05 | [-0.07,0.17] | 0.05 | [-0.07,0.17] | –0.00 | [-0.14,0.13] | 0.02 | [-0.11,0.16] |
| Short-range downward | –0.02 | [-0.13,0.08] | –0.02 | [-0.13,0.08] | 0.01 | [-0.12,0.13] | 0.00 | [-0.12,0.13] |
| Long-range upward | –0.06 | [-0.24,0.12] | –0.07 | [-0.24,0.10] | –0.05 | [-0.24,0.14] | –0.07 | [-0.26,0.12] |
| Short-range upward | 0.08 | [-0.06,0.22] | 0.08 | [-0.05,0.22] | 0.12 | [-0.03,0.27] | 0.11 | [-0.04,0.27] |
| Wave 1 health |  |  |  |  |  |  |  |  |
| Poor self-rated health | 0.22^***^ | [0.13,0.30] | 0.22^***^ | [0.13,0.30] | 0.23^**^ | [0.07,0.40] | 0.18^*^ | [0.02,0.34] |
| Chronic health condition | ––––– | ––––––––– | ––––– | ––––––––– | ––––– | ––––––––– | 0.22 | [-0.03,0.46] |
| Obese | ––––– | ––––––––– | ––––– | ––––––––– | ––––– | ––––––––– | 0.43^***^ | [0.26,0.60] |
| *Interactions* terms |  |  |  |  |  |  |  |  |
| Origin weight | ––––– | ––––––––– | –0.62^**^ | [-1.08,-0.16] | ––––– | ––––––––– | ––––– | ––––––––– |
| Long-range downward | ––––– | ––––––––– | ––––– | ––––––––– | 0.22^*^ | [0.03,0.41] | 0.22^*^ | [0.02,0.41] |
| Short-range downward | ––––– | ––––––––– | ––––– | ––––––––– | 0.07 | [-0.14,0.28] | –0.09 | [-0.33,0.15] |
| Long-range upward | ––––– | ––––––––– | ––––– | ––––––––– | –0.07 | [-0.33,0.18] | –0.05 | [-0.30,0.21] |
| Short-range upward | ––––– | ––––––––– | ––––– | ––––––––– | –0.17 | [-0.42,0.08] | –0.16 | [-0.41,0.10] |
| AIC | 6868.64 |  | 6862.12 |  | 6869.24 |  | 6680.91 |  |
| BIC | 6973.33 |  | 6972.63 |  | 6997.19 |  | 6819.95 |  |
| Observations | 3354 |  | 3354 |  | 3354 |  | 3353 |  |

*Notes:* * p < 0.05, ** p < 0.01, *** p < 0.001.

**Table S3:** Descriptive statistics of CMR components Wave 5

| *Variable* | Mean | SD | Min | Max |
| --- | --- | --- | --- | --- |
| (1) Low density lipoprotein Cholesterol | 105.78 | 31.92 | 9.00 | 332.00 |
| (2) Glucose (Glucose MG/DL) | 95.22 | 35.63 | 41.00 | 618.00 |
| (3) Inflammation (C-reactive protein (CRP)) | 4.01 | 6.26 | 0.15 | 118.00 |
| (4.1) Cardiovascular (systolic blood pressure) | 124.06 | 15.81 | 73.00 | 205.00 |
| (4.2) Cardiovascular (diastolic blood pressure) | 80.01 | 11.18 | 31.00 | 161.00 |
| (4.3) Cardiovascular (resting heart rate) | 74.98 | 12.31 | 40.00 | 135.00 |

**Table S4:** Descriptive statistics

| *Variable* | Mean | SD | Min | Max |
| --- | --- | --- | --- | --- |
| Age at Wave 1 | 14.99 | 1.72 | 12 | 18 |
| Sex (male=1) | 0.39 | 0.49 | 0 | 1 |
| *Race/ethnicity (ref.=White non-Hispanic)* |  |  |  |  |
| Hispanic | 0.12 | 0.33 | 0 | 1 |
| Black non-Hispanic | 0.19 | 0.40 | 0 | 1 |
| Asian | 0.05 | 0.22 | 0 | 1 |
| Other | 0.01 | 0.08 | 0 | 1 |
| *Socioeconomic position variables* |  |  |  |  |
| Parental income, thousands Wave 1 | 52.02 | 61.36 | 0 | 999 |
| Household income Wave 5 | 9.31 | 2.76 | 1 | 13 |
| Personal income Wave 5 | 7.19 | 3.26 | 1 | 13 |
| Educational attainment Wave 5 | 8.61 | 3.30 | 1 | 16 |
| Occupational attainment Wave 5 | 63.60 | 24.10 | 1 | 100 |
| *Wave 1 health* |  |  |  |  |
| Poor self-rated health | 0.30 | 0.46 | 0 | 1 |
| Chronic health condition | 0.02 | 0.16 | 0 | 1 |
| BMI | 22.30 | 4.41 | 11.92 | 53.80 |
| Obese | 0.07 | 0.24 | 0 | 1 |
| *Wave 5 medication use* |  |  |  |  |
| Diabetic | 0.05 | 0.21 | 0 | 1 |
| Hyperlipidemic | 0.04 | 0.19 | 0 | 1 |
| Inflammatory | 0.31 | 0.46 | 0 | 1 |
| Hypertensive | 0.09 | 0.29 | 0 | 1 |

*Notes:* number of observations =5276, except for income measures n=4110 and occupational attainment n=4410

**Table S5:** Accounting for Wave 5 educational and occupational attainment

|  | Model 1 | | Model 2 | |
| --- | --- | --- | --- | --- |
|  | β | [CI95] | β | [CI95] |
| Intercept | 0.95^***^ | [0.93,0.97] | 0.95^***^ | [0.93,0.97] |
| *Immobile income quintiles* |  |  |  |  |
| 1^st^ lowest | 0.12^*^ | [0.02,0.23] | 0.22^***^ | [0.12,0.32] |
| 2^nd^ lowest | 0.03 | [-0.07,0.13] | 0.06 | [-0.04,0.16] |
| Middle | –0.03 | [-0.11,0.04] | –0.04 | [-0.12,0.04] |
| 4^th^ highest | –0.05 | [-0.13,0.03] | –0.10^*^ | [-0.19,-0.01] |
| 5^th^ highest | –0.07 | [-0.16,0.02] | –0.14^**^ | [-0.23,-0.05] |
| Origin weight | 0.74 | [-0.29,1.77] | 0.65^*^ | [0.13,1.16] |
| *Income mobility* |  |  |  |  |
| Long-range downward | –0.01 | [-0.10,0.09] | –0.02 | [-0.11,0.08] |
| Short-range downward | 0.01 | [-0.10,0.11] | 0.00 | [-0.10,0.11] |
| Long-range upward | –0.03 | [-0.20,0.14] | –0.05 | [-0.22,0.12] |
| Short-range upward | 0.06 | [-0.08,0.19] | 0.06 | [-0.08,0.19] |
| Poor self-rated health Wave 1 | 0.06 | [-0.05,0.18] | 0.10 | [-0.01,0.21] |
| *Interactions terms* |  |  |  |  |
| Long-range downward | 0.19^*^ | [0.03,0.35] | 0.17^*^ | [0.01,0.33] |
| Short-range downward | –0.00 | [-0.20,0.19] | –0.01 | [-0.20,0.19] |
| Long-range upward | 0.04 | [-0.13,0.22] | 0.03 | [-0.15,0.21] |
| Short-range upward | –0.08 | [-0.26,0.11] | –0.09 | [-0.27,0.10] |
| *Educational attainment Wave 5 ref.=lowest* |  |  |  |  |
| 2^nd^ lowest | –0.27^**^ | [-0.44,-0.09] | ––––– | ––––––––– |
| Middle | –0.29^***^ | [-0.45,-0.13] | ––––– | ––––––––– |
| 4^th^ highest | –0.50^***^ | [-0.68,-0.33] | ––––– | ––––––––– |
| 5^th^ highest | –0.56^***^ | [-0.73,-0.39] | ––––– | ––––––––– |
| *Occupational attainment Wave 5 ref.=lowest* |  |  |  |  |
| 2^nd^ lowest | ––––– | ––––––––– | 0.05 | [-0.05,0.15] |
| Middle | ––––– | ––––––––– | 0.02 | [-0.08,0.12] |
| 4^th^ highest | ––––– | ––––––––– | –0.05 | [-0.15,0.05] |
| 5^th^ highest | ––––– | ––––––––– | –0.10 | [-0.22,0.01] |
| Adjusted R^2^ | 0.125 |  | 0.112 |  |
| Observations | 5276 |  | 5276 |  |

*Notes:* * p < 0.05, ** p < 0.01, *** p < 0.001.

**Table S6:** Accounting for specific medications use

|  | Model 1 | | Model 2 | |
| --- | --- | --- | --- | --- |
|  | β | [CI95] | β | [CI95] |
| Intercept | 0.93^***^ | [0.91,0.95] | 0.93^***^ | [0.91,0.95] |
| *Immobile income quintiles* |  |  |  |  |
| 1^st^ lowest | 0.22^***^ | [0.13,0.31] | 0.22^***^ | [0.13,0.31] |
| 2^nd^ lowest | 0.06 | [-0.03,0.16] | 0.07 | [-0.02,0.16] |
| Middle | –0.03 | [-0.11,0.05] | –0.03 | [-0.11,0.05] |
| 4^th^ highest | –0.09^*^ | [-0.18,-0.00] | –0.09^*^ | [-0.18,-0.00] |
| 5^th^ highest | –0.16^***^ | [-0.24,-0.07] | –0.16^***^ | [-0.24,-0.08] |
|  |  |  |  |  |
| Origin weight | 0.63^*^ | [0.14,1.12] | 0.64^**^ | [0.17,1.11] |
| Income mobility: |  |  |  |  |
| Long-range downward | 0.05 | [-0.04,0.14] | –0.01 | [-0.10,0.09] |
| Short-range downward | 0.00 | [-0.08,0.08] | –0.01 | [-0.11,0.10] |
| Long-range upward | –0.03 | [-0.19,0.14] | –0.05 | [-0.21,0.12] |
| Short-range upward | 0.03 | [-0.10,0.16] | –0.05 | [-0.21,0.12] |
| Poor self-rated health Wave 1 | 0.11^***^ | [0.04,0.17] | 0.05 | [-0.08,0.18] |
| *Interactions terms* |  |  |  |  |
| Long-range downward | ––––– | ––––––––– | 0.19^*^ | [0.03,0.35] |
| Short-range downward | ––––– | ––––––––– | –0.01 | [-0.11,0.10] |
| Long-range upward | ––––– | ––––––––– | 0.06 | [-0.11,0.24] |
| Short-range upward | ––––– | ––––––––– | –0.06 | [-0.24,0.12] |
| *Medication use:* |  |  |  |  |
| Diabetic | 0.73^***^ | [0.59,0.87] | 0.73^***^ | [0.59,0.87] |
| Hyperlipidemic | 0.09 | [-0.07,0.24] | 0.09 | [-0.06,0.24] |
| Inflammatory | 0.10^**^ | [0.04,0.16] | 0.10^**^ | [0.04,0.16] |
| Hypertensive | 0.39^***^ | [0.29,0.49] | 0.38^***^ | [0.29,0.48] |
| Adjusted R^2^ | 0.156 |  | 0.156 |  |
| Observations | 5188 |  | 5188 |  |

*Notes:* * p < 0.05, ** p < 0.01, *** p < 0.001, all models include additional

controls as in Model 3 in Table 1.

**Table S7:** Models with household income Wave 5

|  | Model 1 | | Model 2 | |
| --- | --- | --- | --- | --- |
|  | β | [CI95] | β | [CI95] |
| Intercept | 0.96^***^ | [0.94,0.98] | 0.95^***^ | [0.93,0.98] |
| *Immobile income quintiles* |  |  |  |  |
| 1^st^ lowest | 0.25^***^ | [0.16,0.35] | 0.25^***^ | [0.16,0.35] |
| 2^nd^ lowest | 0.11^*^ | [0.03,0.20] | 0.11^*^ | [0.02,0.20] |
| Middle | –0.09^*^ | [-0.18,-0.00] | –0.09^*^ | [-0.18,-0.00] |
| 4^th^ highest | –0.09 | [-0.18,0.00] | –0.09 | [-0.18,0.00] |
| 5^th^ highest | –0.18^***^ | [-0.27,-0.10] | –0.19^***^ | [-0.27,-0.10] |
| Origin weight | 0.51^**^ | [0.18,0.84] | 0.52^**^ | [0.19,0.85] |
| *Income mobility*: |  |  |  |  |
| Long-range downward | 0.06 | [-0.01,0.14] | 0.01 | [-0.08,0.10] |
| Short-range downward | –0.01 | [-0.11,0.09] | –0.03 | [-0.14,0.08] |
| Long-range upward | –0.01 | [-0.15,0.13] | –0.01 | [-0.16,0.13] |
| Short-range upward | 0.00 | [-0.12,0.12] | 0.00 | [-0.12,0.13] |
| Poor self-rated health Wave 1 | 0.12^***^ | [0.06,0.19] | 0.08 | [-0.02,0.19] |
| *Interactions terms* |  |  |  |  |
| Long-range downward | ––––– | ––––––––– | 0.17^*^ | [0.01,0.33] |
| Short-range downward | ––––– | ––––––––– | 0.08 | [-0.11,0.27] |
| Long-range upward | ––––– | ––––––––– | –0.02 | [-0.19,0.15] |
| Short-range upward | ––––– | ––––––––– | –0.01 | [-0.22,0.20] |
| Adjusted R^2^ | 0.111 |  | 0.111 |  |
| Observations | 5276 |  | 5276 |  |

*Notes:* * p < 0.05, ** p < 0.01, *** p < 0.001, all models include additional

controls as in Model 3 in Table 1.
